# Towards a ‘resolution limit’ for DW‐MRI tumor microstructural models: A simulation study investigating the feasibility of distinguishing between microstructural changes

**DOI:** 10.1002/mrm.27551

**Published:** 2018-10-19

**Authors:** Damien J. McHugh, Penny L. Hubbard Cristinacce, Josephine H. Naish, Geoffrey J. M. Parker

**Affiliations:** ^1^ Division of Informatics, Imaging and Data Sciences The University of Manchester Manchester United Kingdom; ^2^ CRUK and EPSRC Cancer Imaging Centre in Cambridge and Manchester United Kingdom; ^3^ Bioxydyn Ltd. Manchester United Kingdom

**Keywords:** diffusion‐weighted MRI, microstructural changes, optimum design, resolution limit, tumor microstructure

## Abstract

****Purpose**:**

To determine the feasibility of extracting sufficiently precise estimates of cell radius, *R*, and intracellular volume fraction, *f*_*i*_, from DW‐MRI data in order to distinguish between specific microstructural changes tissue may undergo, specifically focusing on cell death in tumors.

****Methods**:**

Simulations with optimized and non‐optimized clinical acquisitions were performed for a range of microstructures, using a two‐compartment model. The ability to distinguish between (i) cell shrinkage with cell density constant, mimicking apoptosis, and (ii) cell size constant with cell density decreasing, mimicking loss of cells, was evaluated based on the precision of simulated parameter estimates. Relationships between parameter precision, SNR, and the magnitude of specific parameter changes, were used to infer SNR requirements for detecting changes.

****Results**:**

Accuracy and precision depended on microstructural properties, SNR, and the acquisition protocol. The main benefit of optimized acquisitions tended to be improved accuracy and precision of *R*, particularly for small cells. In most cases considered, higher SNR was required for detecting changes in *R* than for changes in *f*_*i*_.

****Conclusions**:**

Given the relative changes in *R* and *f*_*i*_ due to apoptosis, simulations indicate that, for a range of microstructures, detecting changes in *R* require higher SNR than detecting changes in *f*_*i*_, and that such SNR is typically not achieved in clinical data. This suggests that if apoptotic cell size decreases are to be detected in clinical settings, improved SNR is required. Comparing measurement precision with the magnitude of expected biological changes should form part of the validation process for potential biomarkers.

## 1 INTRODUCTION

The motivation for using microstructural models in the analysis of diffusion‐weighted (DW) MRI data stems from the potential for charactering tissue microstructure more specifically than with phenomenological indices. For example, microstructural models have found applications in characterizing neural tissue in terms of parameters such as neurite diameter, packing density/volume fraction, and compartment diffusivities, as opposed to the mean diffusivity and fractional anisotropy,^1^, ^2^, ^3^, ^4^, ^5^ and in characterizing tumor tissue in terms of cell size, volume fraction, and compartment diffusivities, as opposed to the apparent diffusion coefficient (ADC).^6^, ^7^, ^8^, ^9^

A specific application of these models for cancer research is in interpreting DW signal changes more specifically. For example, instead of simply observing an increase or decrease in tumor ADC, estimates of model parameters potentially provide information about the cellular‐level changes which underlie ADC changes. In particular, in cases where different microstructural changes result in similar changes in ADC, the use of biophysical models may allow these different changes to be distinguished. Such changes could, for example, relate to different ways tumors may respond to therapy. For example, tumor cells reducing in size without an overall change in the number of cells, (for example, in cells undergoing apoptotic cell shrinkage but before phagocytosis^10^) can cause an ADC increase,^11^ as can a situation where cell size does not change but the number of cells, and therefore cell density, decreases.^12^ While ADC measurements may be sensitive to such microstructural changes, ADC on its own cannot distinguish between them. However, if tissue properties such as cell size and volume fraction can be estimated directly using microstructural models, the two scenarios above can potentially by distinguished. In such a case, the utility of microstructural models depends upon their ability to resolve ambiguities in ADC interpretation.

The extent to which this can be achieved depends on the accuracy and precision with which model parameters can be estimated, as well as the magnitude of specific biological changes. For example, if typical changes in cell size are smaller than the precision of cell size estimates, such changes will not be detected. This is especially important when considering tumor response to therapy, as different forms of cell death lead to different changes in cell size; cell shrinkage is a hallmark of apoptosis,^13^, ^14^ while cell swelling is linked to necrosis.^13^ As discussed in more detail below, a change in cell size associated with apoptosis is ∼1–4 μm, with a corresponding change in volume fraction of ∼0.1–0.5. While biophysical models have previously been used to infer sensitivity to apoptotic cell shrinkage, it should be noted that this was based on decreases observed in the intracellular volume fraction, with no significant change in cell size detected.^6^, ^15^

This work uses simulations to evaluate the accuracy and precision of parameter estimates from a simple two‐compartment model of tumor tissue, and assesses the ability to distinguish between specific microstructural changes relevant to tumors. This assessment uses ideas similar to the resolution limit recently described for axonal diameter estimates,^16^, ^17^ here focussing on the signal‐to‐noise ratio (SNR) required for obtaining sufficiently precise estimates such that apoptotic cell shrinkage can be detected. Optimized and non‐optimized acquisitions are considered, along with the influence of maximum gradient strength. The paper begins by outlining the general simulation methods, then describes the protocol optimization, and the subsequent in silico experiments addressing accuracy, precision, and the resolution limit.

## 2 METHODS

### 2.1 Simulation methods

The normalized pulsed gradient spin‐echo (PGSE) signal, *S*/*S*_0_, was modeled with an analytic expression combining restricted diffusion inside a sphere, *S*_*i*_,^18^ with hindered extracellular diffusion (with the diffusivity reduced by a tortuosity factor), *S*_*e*_,^19^ and T_2_ relaxation,

(1) $$S/S_{0}\;=\;\exp\left(-\mathrm{TE}/T_{2}\right)\left[f_{i}S_{i}\left(R,D_{i},G,\Delta ,\delta \right)+\left(1-f_{i}\right)S_{e}\left(f_{i},D_{e},b\right)\right].$$

Tissue microstructure was therefore characterized in terms of the cell radius, *R*, intracellular volume fraction, *f*_*i*_, intra‐ and extra‐cellular diffusivities, *D*_*i*_ and *D*_*e*_, and T_2_ (assumed to be the same in the intra‐ and extra‐cellular spaces for simplicity, and taken as 125 ms, a median value reported by Oh et al. for meningiomas^20^); note that the model assumes no water exchange between the intra‐ and extra‐cellular spaces. Acquisition protocols were characterized by gradient strength, *G*, duration, δ, separation, Δ, and echo time, TE. One thousand five hundred noisy synthetic signals were generated for each simulated microstructure and acquisition protocol according to Equation (1), with noise added such that the signals were Rician distributed.^21^ SNR was defined based on the *b* = 0 s/mm^2^ signal at TE  = 100 ms. The model (Equation (1)) was fitted to the 1500 noisy signals using maximum likelihood fitting, accounting for Rician noise.^22^ One hundred starting values were used for each fit, and the final parameter estimates were taken as those giving the lowest value of the objective function. A Nelder‐Mead simplex algorithm was used for fitting, and parameters were constrained to the following ranges: 0.1 ≤ *R* (μm) ≤ 25, 0.01 ≤ *f*_*i*_ ≤ 1, 0.1 ≤ *D*_*i*_ (μm^2^/ms) ≤ 3.0, and 0.1 ≤ *D*_*e*_ (μm^2^/ms) ≤ 3.0. Fitting was also performed with *D*_*i*_ and *D*_*e*_ fixed to their ground truth values, to stabilize the fits. As fixing *D*_*i*_ and *D*_*e*_ increased the fit stability, these data were used to calculate accuracy, precision, and resolution limits, after excluding fits with extreme values (within 1% of the fit constraints). Accuracy was calculated as the mean difference between fitted values and the ground truth, and precision was calculated as the standard deviation of the fitted values, that is, the parameter's standard error, SE (taking the fitted values as the parameter's sampling distribution^23^). Low absolute values for accuracy and precision, as defined here, indicate good performance.

### 2.2 Protocol optimization

Optimum PGSE scan parameters (*G*,Δ,δ) are those that maximize or minimize some summary statistic of the signal model's information matrix, *M*.^24^, ^25^ Here, D‐optimum designs are considered, which correspond to scan parameters that maximize the determinant of the information matrix, |*M*|, and can be interpreted as minimizing the volume of ellipsoidal confidence regions for the model parameters.^25^ For non‐linear models, *M* depends on the specific values of the model parameters, meaning here that *D*‐optimum designs vary with *R*, *f*_*i*_, *D*_*i*_ and *D*_*e*_. Optimum designs calculated for specific tissue properties are therefore referred to as *local* optima. In practice, tissue properties are not known a priori, so it is useful to consider optimum designs which do not depend on one given combination of *R*, *f*_*i*_, *D*_*i*_ and *D*_*e*_. These are referred to as *robust* optima, and can be calculated by finding the minimum of the objective function integrated over a distribution of tissue properties. This is approximated by a summation, with the objective function to minimize for robust D‐optimum designs given by^25^:

(2) $$f\;=\;\sum_{i}^{N}-\log(|M_{i}\left|\right),$$

where *N* is the number of combinations of *R*, *f*_*i*_, *D*_*i*_ and *D*_*e*_. Equation (2) was minimized for *N* = 100, with tissue properties sampled from uniform distributions over the following ranges: 5 ≤ *R* (μm) ≤ 20, 0.1 ≤ *f*_*i*_ ≤ 0.74, 0.5 ≤ *D*_*i*_ (μm^2^/ms) ≤ 3, and 0.5 ≤ *D*_*e*_ (μm^2^/ms) ≤ 3. The objective function was minimized using a genetic algorithm (*ga* in MATLAB), with {*G*,Δ,δ} combinations satisfying clinically‐relevant constraints: *b* ≥ 150 s/mm^2^ (to avoid perfusion effects), Δ−δ ≥ T_180_, and Δ + δ ≤ TE−T_*c*_, where T_180_ = 12 ms is the time for the 180^∘^ refocussing pulse and crushers, and T_*c*_ = 13 ms is the combined time for the readout and the time before the first diffusion gradient. As a means of avoiding protocols yielding low SNR measurements, all scans were constrained to have a maximum TE of 100 ms and a maximum *b*‐value of 5000 s/mm^2^, along with a lower SNR limit of 2, calculated for an ‘average’ microstructure, taken as the median of each tissue property over the *N* = 100 combinations. The effect of the maximum gradient strength, *G*_max_, was investigated by performing the optimization separately for three cases, *G*_max_ = {60, 80, 300} mT/m. For each case, the genetic algorithm was run with a population size of 1600 and was repeated three times with different initial populations; the final optimum design was taken from the repeat with the lowest value of the objective function.

In addition to these D‐optimum acquisitions, simulations were also performed using non‐optimized acquisitions. These were designed to match the D‐optimum acquisitions in terms of *G*_max_ and *b*_max_, and consisted of four measurements in the {*G*, Δ} parameter space, with δ fixed, as used, for example, in AxCaliber acquisitions.^1^ All protocols are given in Table 1, and will be referred to as D‐opt_*G*_ and Non‐opt_*G*_, where the subscript represents the maximum gradient strength. For all protocols, each synthetic signal was normalized to a *G* = 0 mT/m signal with the same TE, as done experimentally.^6^

**Table 1:** Parameters for optimized and non‐optimized acquisitions

D‐opt_300_				
*G* (mT/m)	56.4	38.9	297	109
Δ (ms)	23.2	62.2	16.3	82.7
δ (ms)	10.6	24.8	4.28	4.22
D‐opt_80_				
*G* (mT/m)	43.1	44.9	80.0	80.0
Δ (ms)	65.2	77.3	26.1	19.3
δ (ms)	21.7	9.72	14.1	7.25
D‐opt_60_				
*G* (mT/m)	35.9	36.2	60.0	60.0
Δ (ms)	59.4	27.3	79.2	29.6
δ (ms)	27.6	15.3	7.85	17.6
Non‐opt_300_				
*G* (mT/m)	150	150	300	300
Δ (ms)	80.0	20.0	80.0	20.0
δ (ms)	2.65	2.65	2.65	2.65
Non‐opt_80_				
*G* (mT/m)	40.0	40.0	80.0	80.0
Δ (ms)	80.0	20.0	80.0	20.0
δ (ms)	10.2	10.2	10.2	10.2
Non‐opt_60_				
*G* (mT/m)	30.0	30.0	60.0	60.0
Δ (ms)	80.0	20.0	80.0	20.0
δ (ms)	13.5	13.5	13.5	13.5

### 2.3 Accuracy and precision of microstructural properties

Initial simulations investigated how the accuracy and precision of microstructural properties vary with the properties themselves. Here, simulations were performed for 25 microstructures with all combinations of *R* = 4, 7, 10, 13, 16 μm and *f*_*i*_ = 0.12, 0.24, 0.36, 0.48, 0.60; in each case, *D*_*i*_ = 1 μm^2^/ms, *D*_*e*_ = 2 μm^2^/ms, and T_2_ = 125 ms, representing a range of plausible tumor microstructures.^8^, ^20^, ^26^ Each simulation was run with SNR  =  20 and 80, for D‐opt_80_ and Non‐opt_80_, with the accuracy and precision of *R* and *f*_*i*_ evaluated as described above.

### 2.4 Microstructural changes and resolution limit

**Figure 1:**
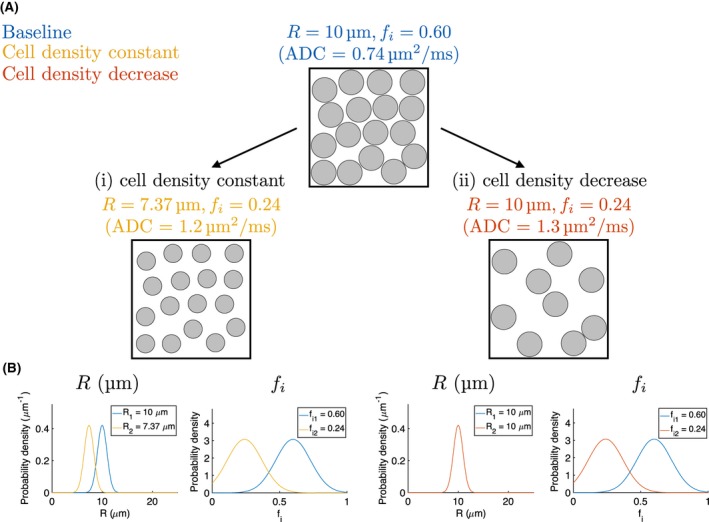
(A) Schematic of microstructural changes. Starting from a ‘baseline’ with *R* = 10 μm and *f*_*i*_ = 0.60, two changes are considered: (i) a 60% decrease in cell volume (*R* → 7.37 μm), mimicking apoptotic cell shrinkage, with corresponding decrease in volume fraction, keeping cell density (number of cells per unit volume) constant; (ii) a decrease in volume fraction with no change in cell size, decreasing cell density, mimicking a loss of cells. Both changes result in an ADC increase, as measured with a typical multi‐*b*‐value clinical acquisition (see text for sequence details). (B) Theoretical Gaussian PDFs illustrating changes in *R* and *f*_*i*_ for changes (i) and (ii), on the left and right, respectively. PDF widths represent the maximum width possible (that is, the precision limit) for successfully detecting the changes, based on a *z*‐test with α = 0.05, assuming equal precision for the ‘baseline’ and ‘change’ estimates; as there is no *R* change in case (ii), the PDF width here is arbitrary. In this example, *D*_*i*_ = 1 μm^2^/ms and *D*_*e*_ = 2 μm^2^/ms, for ‘baseline and ‘change’ microstructures

Simulations were then performed to assess the extent to which specific changes can be detected, in particular looking at the SNR requirements for achieving sensitivity to changes in *R* and *f*_*i*_. This analysis starts with a ‘baseline’ microstructure with *R* = *r*,* f*_*i*_ = *f*,* D*_*i*_ = *di*, and *D*_*e*_ = *de*. Two possible microstructural changes were then considered: (i) a simple mimic of apoptotic cell shrinkage, with a decrease in cell volume of 60%,^14^ decreasing *R* to (0.4^1/3^)*r* with an associated decrease in *f*_*i*_ to 0.4*f*, with cell density, *ρ* = 3*f*_*i*_/(4π*R* ^3^) (that is, the number of cells per unit volume) remaining constant; and (ii) a simple mimic of complete cell death, where *f*_*i*_ decreases to 0.4*f* but *R* remains constant, giving a decrease in ρ. A specific example is shown in Figure 1A, with a baseline of *R* = 10 μm, *f*_*i*_ = 0.60, *D*_*i*_ = 1 μm^2^/ms, and *D*_*e*_ = 2 μm^2^/ms, representing a plausible model of tumor tissue.^8^, ^26^ In this case, change (i) results in *R* decreasing to 7.37 μm with an associated decrease in *f*_*i*_ to 0.24, and change (ii) results in *f*_*i*_ decreasing to 0.24, with *R* unchanged. Here, changes (i) and (ii) would both result in an ADC increase (see Figure 1A), as measured with a typical multi‐*b*‐value clinical protocol (*b* = 150, 300, 500, 800 s/mm^2^ with *G* = 13.2, 18.6, 24.0, 30.4 mT/m, Δ = 32.0 ms and δ = 22.2 ms), but ADC alone cannot distinguish between (i) and (ii). As above, T_2_ was taken as 125 ms, and *D*_*i*_ and *D*_*e*_ were assumed to not change from baseline values. In addition to the specific example in Figure 1, a range of baseline microstructures were investigated, with values within relevant biological ranges: *R* = 7–16 μm, *f*_*i*_ = 0.30–0.60, *D*_*i*_, *D*_*e*_ = 1–2 μm^2^/ms.

Similar to the resolution limit for axonal diameter estimates,^16^ we define the ability to detect a change in a parameter, *p*, in terms of a two sample z‐test, with α = 0.05:

(3) $$\sqrt{{\mathrm{SE}}_{p_{1}}^{2}\;+\;{\mathrm{SE}}_{p_{2}}^{2}}\;\leq \;\frac{\Delta p}{1.96},$$

where ${\mathrm{SE}}_{p_{n}}$ is the standard error on the *n*th estimate of parameter *p*, and $\Delta p\;=\;|p_{2}-p_{1}|$ is the magnitude of the parameter change; *p* = {*R*, *f*_*i*_}, *n* = {1, 2}. This is illustrated in Figure 1B, which shows the theoretical broadest Gaussian probability density functions (PDFs) consistent with resolving the changes. Note that this approach differs from Nilsson et al.,^16^ where the statistical test is based on the change in signal itself. The effect of SNR on ${\mathrm{SE}}_{p_{n}}$ was evaluated using simulations as described above, with SNR  =  20, 50, 80 and 110. Results were extended to a wider range of SNRs by fitting the expression *m*/SNR + *c* (where *m* and *c* are fitted variables) to the calculated resolution limits (left hand side of Equation (3)); fits were stabilized by including a point reflecting the expectation that SE_*p*_→0 as SNR→∞. The intersection of these fitted curves with the relevant detection threshold (right hand side of Equation (3)) allowed inference of the SNR required for detecting a given change.

### 2.5 Influence of percentage cell volume decrease

Simulations were also performed to assess the influence of the percentage cell volume decrease used to mimic apoptosis. The 60% decrease described above was taken from the largest observed volume change in an in vitro study where cells were exposed to the chemotherapy drug cisplatin for 96 hours,^14^ providing a ‘best case’ scenario for detecting Δ*R*. As smaller decreases may be more realistic, a subset of simulations considered changes of 40% and 20%. These simulations were performed for *R* = 10, 16 μm, *f*_*i*_ = 0.60, *D*_*i*_, *D*_*e*_ = 1–2 μm^2^/ms, using D‐opt_80_.

### 2.6 Modeling asynchronous apoptosis

All of the simulations described above model apoptosis by assuming that all cells shrink; that is, assuming apoptosis is synchronous. To investigate the effect of asynchronous apoptosis, simulations were also performed in which a fraction of cells shrink, with the rest remaining the same size. These simulations are described in the Supporting Information.

## 3 RESULTS

### 3.1 Accuracy and precision of microstructural properties

**Figure 2:**
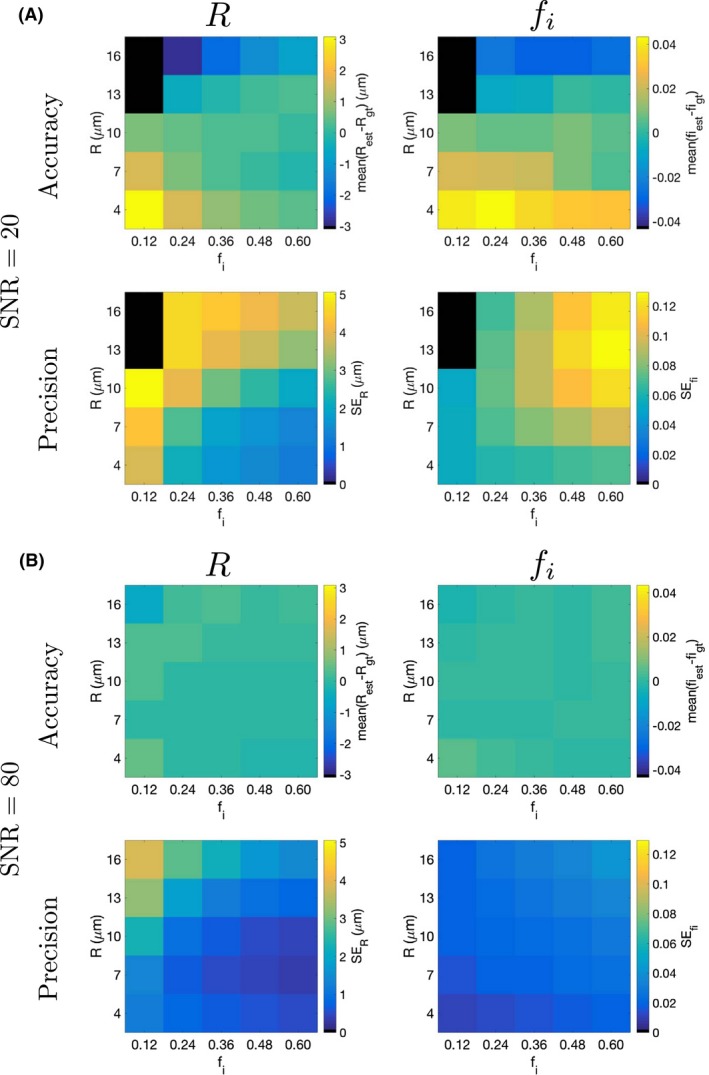
Accuracy and precision of radius and intracellular volume fraction, using D‐opt_80_, plotted as a function of ground truth *R* and *f*_*i*_, for (A) SNR  =  20, and (B) SNR  =  80. For (A), color scales in each panel are based on the maximum absolute value plotted, and the same color scales are used for the equivalent panels in (B). Black points represent cases where more than 50% of the fits resulted in extreme values (within 1% of the fit constraints) for at least one parameter

The dependence of parameter accuracy and precision on microstructural properties is illustrated in Figure 2, where the accuracy and precision of *R* and *f*_*i*_ are plotted for different ground truth combinations, using D‐opt_80_ at SNR  =  20 and 80. Black points represent excluded cases where more than 50% of the fits resulted in extreme values (within 1% of the fit constraints) for at least one parameter; note that this occurs for large cells with low volume fraction, where signal attenuation is greatest and measurements are therefore most affected by the noise floor. At SNR  =  20, *R* and *f*_*i*_ can be under‐ or over‐estimated, depending on the ground truth (Figure 2A). Both *R* and *f*_*i*_ tend to be underestimated at high *R*, and overestimated at low *R*, with the highest accuracy tending to occur when *R* = 10 μm. For a given *R*, accuracy in *R* tends to increase (that is, the magnitude of the difference from the ground truth tends to decrease) as *f*_*i*_ increases, and the largest overestimation occurs for low *R* and low *f*_*i*_, that is, small cells with low volume fraction. Accuracy improves at SNR  =  80, and is typically better than 1% for both parameters, except at low *f*_*i*_ with small and large cells, where accuracy is poorer (<15% for *R* and <5% for *f*_*i*_, Figure 2B). *R* tends to be estimated more precisely (that is, SE_*R*_ is lower) at higher *f*_*i*_, while estimates of *f*_*i*_ tend to be more precise at lower *f*_*i*_. For a given *f*_*i*_, precision in *R* tends to worsen as *R* increases. Similar trends in precision are observed at SNR  =  20 and 80, but precision is better at the higher SNR as expected. The equivalent figure for Non‐opt_80_ is shown in Supporting Information Figure S1 (see Supporting Information), which exhibits very similar trends to those for D‐opt_80_. Similar trends were also observed for D‐opt_60_, but accuracy and precision tended to be slightly worse than for D‐opt_80_ for the majority of the *R* and *f*_*i*_ combinations at SNR  =  20, with smaller differences between the two gradient strengths at SNR  =  80 (data not shown). For D‐opt_300_, the dependence of accuracy and precision on the ground truth exhibited similar trends to those observed at the lower gradient strengths. Accuracy and precision tended to be better with D‐opt_300_ than with D‐opt_80_, except for large cells (*R* = 13, 16 μm), where D‐opt_80_ tended to perform better, most notably in terms of precision in *R*.

**Figure 3:**
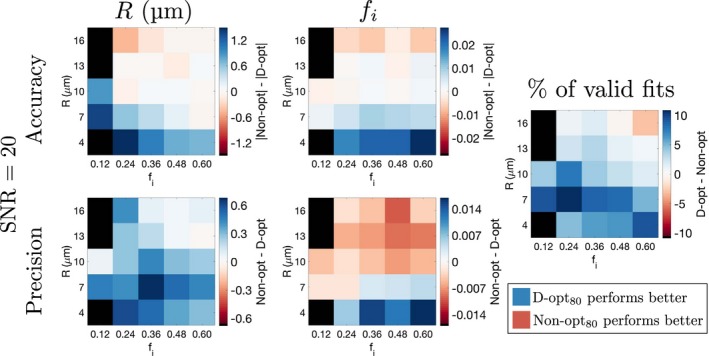
Comparing accuracy and precision of *R* and *f*_*i*_ estimates from D‐opt_80_ and Non‐opt_80_. Differences in accuracy and precision between the two acquisitions are plotted as a function of ground truth *R* and *f*_*i*_, for SNR  =  20. For each panel, black points represent cases where more than 50% of the fits resulted in extreme values (within 1% of the fit constraints) for at least one parameter, for either acquisition. The rightmost column plots the difference in the percentage of valid fits between the two acquisitions. In all cases, the definitions of differences are such that positive regions (blue) correspond to parts of the parameter space where D‐opt_80_ performs better than Non‐opt_80_

**Figure 4:**
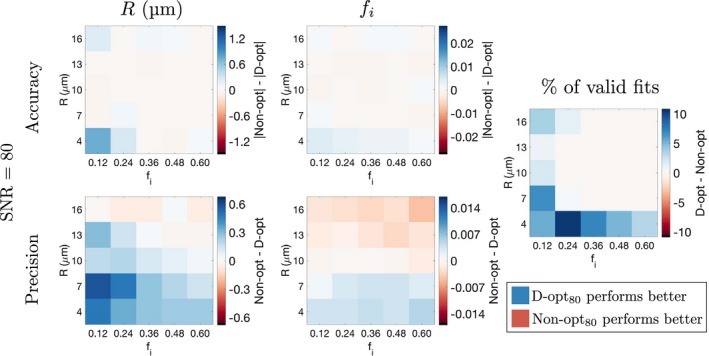
As Figure 3 but for SNR  =  80. The color scales used here match those of the equivalent panels in Figure 3

The difference in accuracy and precision between D‐opt_80_ and Non‐opt_80_, as a function of the ground truth *R* and *f*_*i*_, is shown for SNR  =  20 (Figure 3) and SNR  =  80 (Figure 4); differences in absolute values are used for comparing accuracies, as the accuracy metric can be positive or negative. In addition, the rightmost column in Figures 3 and 4 plots the difference in the percentage of valid fits (those not within 1% of the constraints) between the two acquisitions. In all panels, differences are defined such that positive regions (blue) correspond to parts of the parameter space where D‐opt_80_ performs better than Non‐opt_80_. At SNR  =  20, D‐opt_80_ tends to yield better accuracy and precision in *R* at low ground truth *R*, and generally has a higher percentage of valid fits (blue regions in Figure 3). However, Non‐opt_80_ offers better *f*_*i*_ precision at higher *R* (red regions in *f*_*i*_ precision panel in Figure 3). At SNR  =  80, the two acquisitions tend to perform more similarly, as shown by the white regions throughout Figure 4. Here, D‐opt_80_ offers better precision in *R* and *f*_*i*_ at low *R*, while there is still a tendency for Non‐opt_80_ to yield better *f*_*i*_ precision at higher *R*.

Similar trends to those described above were also found when comparing D‐opt_60_ and Non‐opt_60_. Again, the main benefit of the optimized acquisition was improved accuracy and precision of *R* at low ground truth *R*, with the non‐optimized acquisition providing better *f*_*i*_ precision at higher *R*. In contrast to the comparison for *G*_max_ = 80 mT/m, Non‐opt_60_ also provided slightly better precision in *R* for large cells.

### 3.2 Microstructural changes and resolution limit

**Figure 5:**
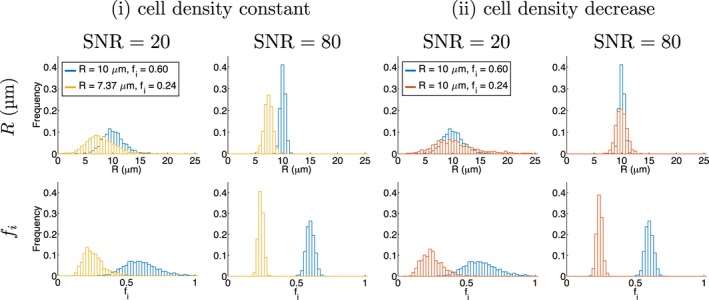
Parameter histograms from simulations using D‐opt_80_, for the microstructures in Figure 1, at SNRs of 20 and 80

**Figure 6:**
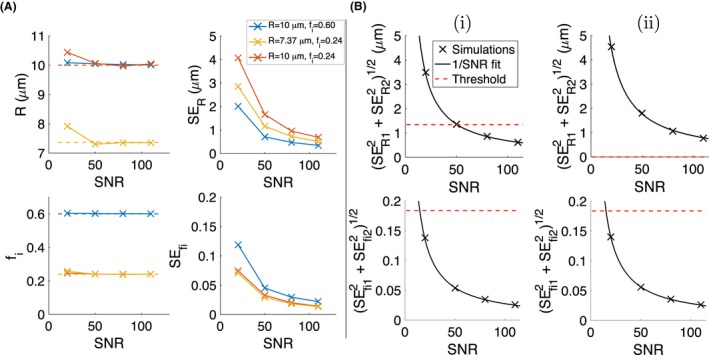
(A) *R* and *f*_*i*_ accuracy (left, dashed lines indicate ground truth) and precision (right) as a function of SNR, for the three microstructures. (B) ‘Resolution limit’ (left hand side of Equation (3)) for *R* and *f*_*i*_, as a function of SNR, for changes (i) and (ii). Black lines show 1/SNR fit to the simulation‐derived resolution limits, and red dashed lines show the threshold for detecting change, calculated from the right hand side of Equation (3). Note that there is no change in *R* for (ii), so there is no threshold for detecting change

Figure 5 plots *R* and *f*_*i*_ histograms from simulations for the microstructural changes illustrated in Figure 1, using D‐opt_80_. At SNR  =  20, precision depends on the microstructure, indicating that the assumption of equal precision for ‘baseline’ and ‘change’ estimates used in Figure 1B does not always hold. This is most apparent for the *R* = 10 μm, *f*_*i*_ = 0.60 microstructure (blue histograms), where *f*_*i*_ precision is poorer and *R* precision is better than the other microstructures. This is consistent with the trends in absolute precision discussed above, and is also reflected in Figure 6A (right column), where precision is plotted against SNR for the three microstructures. Accuracy is plotted in Figure 6A (left column), where error bars have been omitted due to the large overlap for different microstructures; note that the error bars would reflect the precision values in Figure 6A (right column). Qualitatively, the overlap in *R* histograms for (i) at SNR  =  20 suggests that precision is insufficient to detect this change, implying that changes (i) and (ii) cannot be distinguished. As SNR increases, the variation in precision between microstructures tends to decrease, and the histograms indicate that at SNR  =  80 the two radii for change (i) are better resolved. This is quantified in Figure 6B, where the ‘resolution limit’ (black crosses, left hand side of Equation (3)) is plotted against SNR, along with the threshold for detection (red dashed lines, right hand side of Equation (3)). As expected from the histograms, the resolution limit for *R* at SNR  =  20 exceeds the threshold, while at SNR  =  80 it is sufficient to detect the change. For changes (i) and (ii), SNR  =  20 is sufficient to detect the changes in *f*_*i*_.

The resolution limits are well‐described by the 1/SNR relationship (Figure 6B, black lines), with R^2^ > 0.997 in each case. This fit was performed based on the three resolution limits above SNR  =  20, as including this point in some cases resulted in very poor fits. This was hypothesized to be due to unstable estimates of SE_*R*_ and ${\mathrm{SE}}_{f_{i}}$ at low SNR, where a larger proportion of fits can return extreme values (within 1% of the fit constraints). While this was not the case for all microstructures, for consistency, the resolution limit for SNR  =  20 was excluded from the 1/SNR fit throughout. Using this fit to interpolate between and extrapolate beyond the four SNRs simulated suggests that SNRs of 51 and 15 are needed for detecting the changes in *R* and *f*_*i*_, respectively (see intersections of black solid lines with red dashed lines in Figure 6B). Taken together, this suggests that given the relative changes in *R* and *f*_*i*_ due to apoptosis, sensitivity to Δ*R* requires ∼3‐fold higher SNR than sensitivity to *f*_*i*_. Note that this result comes from considering the single baseline microstructure and specific changes illustrated in Figure 1.

**Figure 7:**
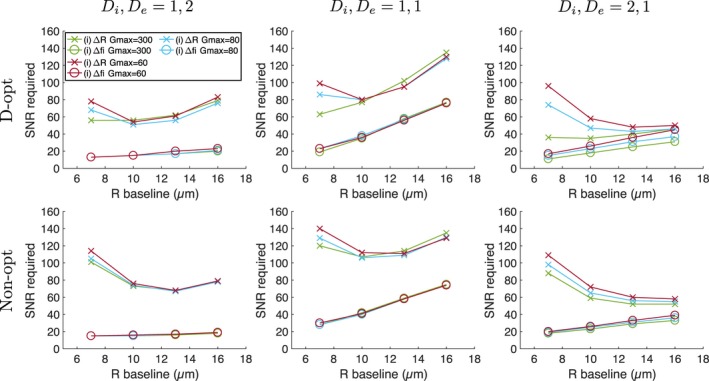
SNR required for detecting Δ*R* (crosses) and Δ*f*_*i*_ (circles) for change (i), as a function of ‘baseline’ cell size. Results are shown for three combinations of *D*_*i*_ and *D*_*e*_ (columns, units: μm^2^/ms), for D‐opt and Non‐opt acquisitions (rows). In each plot, different colors represent acquisitions with different *G*_max_

To determine how this sensitivity‐SNR relationship depends on the microstructural properties themselves, the above analysis was conducted for a range of baseline cell sizes, *R* = 7, 10, 13, 16 μm, in each case considering three combinations of diffusivities, with *D*_*i*_ less than, equal to, or greater than *D*_*e*_: *D*_*i*_, *D*_*e*_ = {1,2}, {1,1}, {2,1}μm^2^/ms. For each case, the resolution limit for changes (i) and (ii) were obtained from simulations using all acquisitions (Table 1), allowing the effect of protocol to be investigated. As above, the SNR required for detecting the changes was estimated from the intersection of the detection threshold and the curve fitted to the resolution limits. R^2^ values for these fits ranged from 0.843 to >0.999. Figure 7 plots the SNR required for detecting Δ*R* and Δ*f*_*i*_ in change (i) as a function of baseline *R*, for the three diffusivity combinations, for all acquisitions. As the SNR required for detecting Δ*f*_*i*_ in change (ii) was similar to that for change (i), these points were omitted from the plots for clarity. In all scenarios a higher SNR is required for detecting Δ*R* than for Δ*f*_*i*_, consistent with the initial observations made above, although there is variation depending on specific cell sizes and diffusivities. For Δ*R*, the dependence on baseline cell size is a result of two effects: first, as baseline *R* increases, the absolute value of Δ*R* increases, which lowers SNR requirements; but second, there is a tendency for precision in *R* to worsen as *R* increases, thereby increasing SNR requirements. For *D*_*i*_, *D*_*e*_ = 1, 2 μm^2^/ms, SNRs for detecting Δ*f*_*i*_ show little variation with baseline cell size, while for the other diffusivities the required SNR tends to increase with cell size; this is most apparent for *D*_*i*_, *D*_*e*_ = 1, 1 μm^2^/ms. The required SNRs for detecting the two parameter changes become similar when *D*_*i*_, *D*_*e*_ = 2, 1 μm^2^/ms and cell sizes are relatively large (Figure 7, right column). The main benefit of using optimized over non‐optimized acquisitions tends to occur for detecting Δ*R* when cells are small, with the D‐opt protocols yielding lower required SNRs. For Δ*R* with larger cells, and for sensitivity to *f*_*i*_ generally, there are not clear benefits in using D‐opt protocols, and in some cases the non‐optimized protocol yields lower required SNRs. For optimized and non‐optimized protocols, using *G*_max_ = 80 mT/m offers negligible benefit over *G*_max_ = 60 mT/m for detecting Δ*f*_*i*_, while for Δ*R* there are slight advantages, which are generally greater when baseline *R* is lower. The advantage of *G*_max_ = 300 mT/m tends to be with the optimized protocol for small cell sizes. Note that D‐opt_300_ gives the measurement with the shortest TE (compared with D‐opt_60_ and D‐opt_80_), suggesting an SNR benefit from higher *G*_max_.

**Figure 8:**
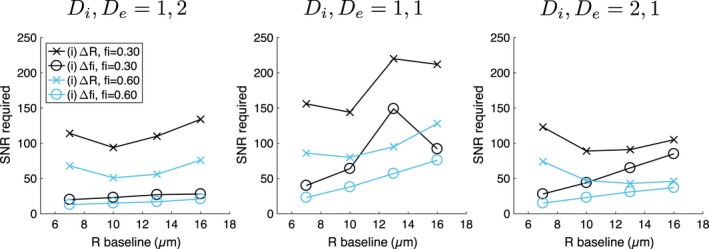
SNR required for detecting Δ*R* (crosses) and Δ*f*_*i*_ (circles) for change (i), as a function of ‘baseline’ cell size and intracellular volume fraction. Results are shown for three combinations of *D*_*i*_ and *D*_*e*_ (columns, units: μm^2^/ms), for baseline *f*_*i*_ values of 0.30 (black lines) and 0.60 (blue lines). The D‐opt_80_ protocol was used in all cases, with the blue lines identical to those in the top row of Figure 7

To investigate the effect of baseline *f*_*i*_, the D‐opt_80_ simulations above were run with a baseline *f*_*i*_ = 0.30, with changes (i) and (ii) leading to *f*_*i*_ = 0.12. Figure 8 plots the SNR required for detecting Δ*R* and Δ*f*_*i*_ in change (i) as a function of baseline *R*, for the three diffusivity combinations, for baseline *f*_*i*_ values of 0.30 (black lines) and 0.60 (blue lines). As above, the SNR required for detecting Δ*f*_*i*_ in changes (i) and (ii) were similar, so the latter points were omitted from the plots. For detecting Δ*f*_*i*_, two competing factors determine the dependence on baseline *f*_*i*_: first, the absolute difference in intracellular volume fractions between ‘baseline’ and ‘change’ cases is lower when baseline *f*_*i*_ is lower (0.30−0.12 = 0.18 compared with 0.60–0.24 = 0.36), which increases SNR requirements; second, *f*_*i*_ precision tends to be better at lower *f*_*i*_, which decreases SNR requirements. Higher SNRs are needed for *f*_*i*_ = 0.30 than for *f*_*i*_ = 0.60 (black lines in Figure 8 are above the corresponding blue lines), suggesting that the former factor dominates, for the cell sizes and diffusivities considered. Higher SNRs are also needed for detecting Δ*R* when *f*_*i*_ = 0.30 than when *f*_*i*_ = 0.60, due to the tendency for *R* precision to worsen at lower *f*_*i*_. Overall, these results suggest that achieving sensitivity to the microstructural changes considered is more difficult for tumor tissue with a lower *f*_*i*_ than for a higher *f*_*i*_.

### 3.3 Influence of percentage cell volume decrease

**Figure 9:**
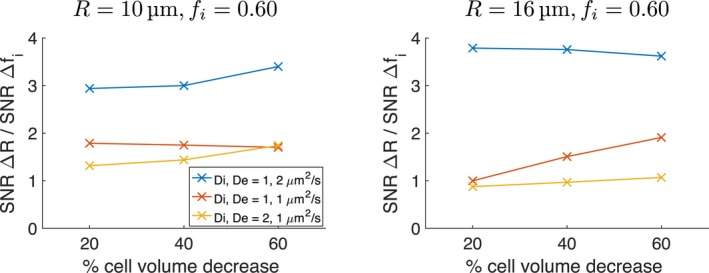
Ratio of SNRs required for detecting Δ*R* and Δ*f*_*i*_, from changes (i) and (ii) respectively, as a function of the simulated percentage cell volume decrease. Results are shown for three combinations of *D*_*i*_ and *D*_*e*_ (colors), for two baseline microstructures (left and right). The D‐opt_80_ protocol was used in all cases

Figure 9 plots the ratio of SNRs required for detecting Δ*R* and Δ*f*_*i*_, from changes (i) and (ii) respectively, as a function of the simulated percentage cell volume decrease, for different microstructures. As the magnitude of Δ*f*_*i*_ decreases as this percentage decreases, the SNR required to detect Δ*f*_*i*_ increases; that is, a higher SNR is needed to detect Δ*f*_*i*_ when there is a 20% cell volume decrease than when there is a 60% cell volume decrease. However, this does not significantly affect the relative thresholds for Δ*R* and Δ*f*_*i*_, because a higher SNR is also needed to detect the correspondingly smaller change in cell size. As such, the tendency is for the SNR thresholds for both parameters to increase as the percentage cell volume decrease gets smaller.

### 3.4 Modeling asynchronous apoptosis

As asynchronous apoptosis was modeled with a bimodal cell radius distribution, while a single radius model (Equation (1)) was fitted to the signals, *R* estimates are biased (Figure S2B). The total restricted volume fraction is estimated accurately (Supporting Information Figure S2B), and the SNR required for detecting Δ*R* can be lower than for Δ*f*_*i*_, when a high proportion of cells remain the same size (Supporting Information Figure S3). These results are described in more detail in the Supporting Information.

## 4 DISCUSSION

These results suggest that using DW‐MRI to detect the subtle changes in cell size which distinguish apoptotic cell shrinkage from simply a reduction in the number of cells is practically challenging in a clinical setting. This is a result of the relatively small absolute change in cell radius during apoptosis, compared with the precision of cell radius estimates at typical SNRs. Initial simulations showed that *R* and *f*_*i*_ precision varies with microstructural properties, suggesting that it may be more feasible to detect changes in tissues with certain characteristics. For example, as *R* precision tends to be better when *f*_*i*_ is high, it may be easier to detect a change in cell size in tumors with a high *f*_*i*_ compared to those with a low *f*_*i*_. This could be practically important in longitudinal studies; for example, if tumor *f*_*i*_ is relatively high at the start of a study, but decreases over time in response to treatment, sensitivity to changes in *R* will decrease over time.

As described above, the 60% decrease in cell volume used here to model apoptosis was taken from the largest observed volume change in an in vitro study^14^; the changes considered in the present study may therefore represent a best‐case scenario, as smaller, and perhaps more realistic changes, will be more difficult to detect. Simulations with 40% and 20% decreases confirmed this, with SNRs for detecting Δ*R* and Δ*f*_*i*_ both increasing as the changes become smaller. While the SNRs required for Δ*R* and Δ*f*_*i*_ were most similar for microstructures with *D*_*i*_, *D*_*e*_ = 2, 1 μm^2^/ms, a higher SNR was needed for Δ*R* in most cases considered. This is qualitatively consistent with microstructural modeling in preclinical experiments (utilizing *G*_max_ = 360 mT/m), where decreases in intracellular volume fraction were detected in tumors undergoing apoptosis, while significant changes in cell size were not.^6^ Although cell shrinkage is a hallmark of apoptosis,^13^ the modeling considered in the present work clearly oversimplifies the apoptotic process, as other morphological changes such as cell shape alterations^27^ have not been considered. Subsequent events such as phagocytosis and tumor cell repopulation have also not been considered, though they would influence the microenvironment; indeed, post‐apoptotic repopulation has been proposed as a potential explanation for an observed lack of ADC change in tumors undergoing apoptosis.^28^ Moreover, apoptosis is known to be asynchronous,^27^ suggesting that even if apoptotic volume decreases can be detected, imaging at a single time point will not reflect the overall level of apoptosis in a tumor. Also, simulations modeling asynchronous apoptosis indicate that *R* estimates are generally biased when using a single radius model, suggesting that relevant cell radii decreases will not be estimated accurately in this setting (see Supporting Information).

The results here also reflect an idealized scenario in terms of fitting DW‐MRI data, as *D*_*i*_ and *D*_*e*_ are assumed to be known, which will generally not be the case in experimental settings, and unchanging, which may not be a valid assumption. The problems with placing constraints on compartmental diffusivities, discussed widely in the context of neural tissue modeling,^29^, ^30^ are equally applicable to tumor tissue. The fixing of diffusivities in the current work therefore suggests the results should be interpreted as a best‐case situation, with accuracy and precision likely to be worse in experimental settings, at least where the scanner constraints match those considered here.

Preclinical studies utilizing different models have found different relationships between intra‐ and extra‐cellular diffusivities, with the long‐time limit of the extracellular diffusivity found to be greater^8^ and lower than the intracellular diffusivity.^9^ Different diffusivities have also been found for tumors from different cell lines,^9^ and different fixed values have been used in different studies.^6^, ^7^ The three diffusivity combinations considered here represent plausible scenarios, and highlight how *D*_*i*_ and *D*_*e*_ influence the ability to detect apoptotic cell shrinkage. The lowest required SNR for detecting Δ*R* occurred for large cells where *D*_*i*_, *D*_*e*_ = 2, 1 μm^2^/ms. As such, for a given acquisition protocol and baseline *R*, sensitivity to apoptotic cell shrinkage may be more feasible for a tumor with these characteristics, while it would be less feasible if, for example, *D*_*i*_, *D*_*e*_ = 1, 1  μm^2^/ms. This highlights the importance of diffusivities in determining sensitivity to changes which are of interest in characterizing tumor response to treatment. Although not considered here, this sensitivity would also be influenced by changes in, and differences between, compartmental T_2_ values. While a single T_2_ was used here for simplicity, evidence for different T_2_ values within and outside axons has recently been presented,^31^ which may also be the case in tumor tissue. Incorporating T_2_ estimation in diffusion models may also aid tumor microstructural estimates.^32^

Recent work by Reynaud has also used simulations to investigate the accuracy and precision of microstructural estimates, comparing ground truth microstructures for three preclinical tumor types, using different acquisitions incorporating both PGSE and oscillating gradient spin‐echo (OGSE) measurements.^15^ Diffusivities were estimated along with cell size and volume fraction, which is beneficial practically, as *D*_*i*_ and *D*_*e*_ do not have to be fixed to assumed values which may bias *R* and *f*_*i*_. Moreover, *D*_*i*_ and *D*_*e*_ may be useful biomarkers themselves, providing information about intra‐ and extra‐cellular structures, such as cell nuclear size and collagen fiber density/alignment. Three factors may contribute to the improved fitting in^15^: (a) the use of OGSE measurements, which increase sensitivity to intracellular diffusion^9^; (b) the use of a higher *G*_max_; (c) the use of a relatively high SNR of 120, which was chosen on the basis of preclinical data.^15^ For similar microstructural parameters to those considered in Ref. 15, an SNR of 120 using D‐opt_80_ and the model considered here, with fitting performed without fixing *D*_*i*_ and *D*_*e*_, yields estimates of *R* and *f*_*i*_ with precision at least as good as those reported previously^15^; diffusivity estimates, however, tend to be poorer, with many values reaching the upper constraint. This suggests that using OGSE measurements, and/or high gradient strengths, may benefit *D*_*i*_ and *D*_*e*_ estimates. This comparison initialized fits to the ground truth, for consistency with Ref. 15, whereas this was not done for the other simulations in the present work. In addition to the protocol differences due to hardware used in the two settings, substantially different SNRs of ∼120 and ∼14 have been reported for preclinical^15^ and clinical^7^ studies, respectively. As such, further work is needed to comprehensively compare preclinical and clinical experiments, as well as to compare PGSE and OGSE sequences with the same hardware constraints, similar to the sensitivity analysis performed for axon diameters.^33^

The aim of using optimized acquisitions is to ensure robust parameter estimates, and their importance has been emphasized in a number of quantitative MR applications.^24^, ^34^, ^35^, ^36^ As optimum design frameworks aim to yield estimates with low variance, this is consistent with focussing on parameter precision, which may be more important than accuracy if the goal is to detect changes (with the caveat that the magnitude and direction of any bias is constant across, for example, baseline and post‐therapy measurements). The simulations performed in the present work suggest that while such protocols do offer benefits in a number of cases, they do not always yield estimates that are better than non‐optimized protocols. This may be due to the fact that the optimization has to be performed to cover a range of possible microstructures, as the properties are not known a priori. As such, there will be particular microstructures for which the acquisition is not ideal, with non‐optimized protocols performing better, suggesting that protocol optimization is not guaranteed to be beneficial, at least for a given choice of optimality criteria. Similar observations have been made previously with other MR signal models, with optimized and non‐optimized protocols performing similarly,^3^ and where non‐optimized protocols out‐perform optimized ones in certain regions of the parameter space.^37^ Another important consideration is the optimality criterion used, and it should be noted that many different criteria can be chosen, based on different summary statistics of the information matrix. D‐optimality was chosen here as it is widely used and generates designs which do not depend on the model parameters’ units, unlike other criteria.^25^ However, as it seeks to minimize the total volume of ellipsoidal confidence regions, it can result in small confidence intervals for some parameters but larger ones for others. This may underlie the observation here that the D‐optimum designs tended to benefit *R* estimates more than *f*_*i*_ estimates, and further work could explore the effect of different optimality criteria.

The resolution limit outlined here essentially relies on a comparison of voxel‐wise parameter estimates, which is not generally reflective of how, for example, baseline and post‐treatment scans would be analyzed in a longitudinal study. Unless an approach similar to the functional diffusion maps developed for ADC^11^ is adopted to directly compare voxels, the test would need to be adapted to include estimates from multiple voxels. This has the potential to reduce voxel‐level SNR requirements, but may, as with all whole‐tumor summary statistics, be confounded by intra‐tumor heterogeneity. Also, the z‐test used to the define the resolution limit assumes a Gaussian distribution of parameter values, which, especially at low SNR, is not always valid. As such, further work could look to adapt the approach presented here to a non‐parametric statistical test.

A further limitation in the practical use of these results concerns the applicability of the model (Equation (1)) to actual tumor tissue. The model necessarily simplifies the tumor microenvironment, and does not include important features such as cell nuclei, immune cells, collagen fibers, and vasculature. Nevertheless, similar models have been successfully applied in a number of preclinical^6^, ^8^, ^9^ and clinical^7^, ^32^ in vivo settings, suggesting that microstructural information beyond ADC can be obtained. In general, further experimental work is needed to validate^38^ microstructural models, and to understand the influence of different microstructural properties on the DW‐MRI signal.

## 5 CONCLUSIONS

The accuracy and precision of DW‐MRI microstructure estimates depends on specific microstructural properties as well as SNR and the acquisition protocol used. Given the relative changes in *R* and *f*_*i*_ as a result of apoptosis, simulations indicate that, for PGSE acquisitions and a wide range of microstructures, detecting changes in *R* require higher SNR than detecting changes in *f*_*i*_, and that such SNR is typically not achieved in clinical data. This suggests that if apoptotic cell size decreases are to be detected in clinical settings, improved SNR is required. Understanding the SNR requirements for detecting specific microstructural changes should be considered before planning experimental studies, and, more generally, comparing the magnitude of expected biological changes with the accuracy and precision of measurements should form part of the validation process^38^ for potential biomarkers.

## CONFLICT OF INTEREST

G.J.M. Parker has a shareholding and part time appointment and directorship at Bioxydyn Ltd. which provides diffusion MRI services.

## Supporting information


**FIGURE S1** Accuracy and precision of radius and intracellular volume fraction, using Non‐opt_80_, plotted as a function of ground truth *R* and *f*_*i*_, for (A) SNR  =  20, and (B) SNR  =  80. Black points represent cases where more than 50% of the fits resulted in extreme values (within 1% of the fit constraints) for at least one parameter. Color scales match those in Figure 2 in main text
**FIGURE S2** Effects of having a bimodal cell radius distribution. (A) Schematic of baseline microstructure and changes, where a fraction, *p*, of cells remain the same size, and the rest, 1−*p*, shrink. A specific example with *p* = 0.50 is used in the schematic, with the equations governing the changes shown at the bottom. The case of *p* = 0 corresponds to the simulations in the main text; that is, where all cells shrink. (B) Accuracy of *R* (top row) and *f*_*i*_ (bottom row) for three microstructures (colors), for *p* = 0,0.25, 0.50, 0.75; dashed lines indicate ground truth, with the gold lines for *R* and *f*_*i*_ representing *R*_*b*_ and $f_{i_{b}}$, respectively
**FIGURE S3** SNR required for detecting Δ*R* (crosses) and Δ*f*_*i*_ (circles), from changes (i) and (ii), respectively, as a function of *p*, the fraction of cells remaining the same size
