# Supplementary material for: Towards a ‘resolution limit’ for DW‐MRI tumor microstructural models: A simulation study investigating the feasibility of distinguishing between microstructural changes
Source: Magn Reson Med. 2018 Oct 19;81(4):2288–301. doi: 10.1002/mrm.27551 (PMC6492139; doi:10.1002/mrm.27551)
Supplement: Supplementary file 1 — FIGURE S1 Accuracy and precision of radius and intracellular volume fraction, using Non‐opt80, plotted as a function of ground truth R and f i, for (A) SNR = 20, and (B) SNR = 80. Black points represent cases where more than 50% of the fits resulted in extreme values (within 1% of the fit constraints) for at least one parameter. Color scales match those in Figure 2 in main text FIGURE S2 Effects of having a bimodal cell radius distribution. (A) Schematic of baseline microstructure and changes, where a fraction, p, of cells remain the same size, and the rest, 1−p, shrink. A specific example with p = 0.50 is used in the schematic, with the equations governing the changes shown at the bottom. The case of p = 0 corresponds to the simulations in the main text; that is, where all cells shrink. (B) Accuracy of R (top row) and f i (bottom row) for three microstructures (colors), for p = 0,0.25, 0.50, 0.75; dashed lines indicate ground truth, with the gold lines for R and f i representing R b and fib, respectively FIGURE S3 SNR required for detecting ΔR (crosses) and Δf i (circles), from changes (i) and (ii), respectively, as a function of p, the fraction of cells remaining the same size [file MRM-81-2288-s001.pdf]

Supporting information

Accuracy and precision for Non-opt$_{80}$

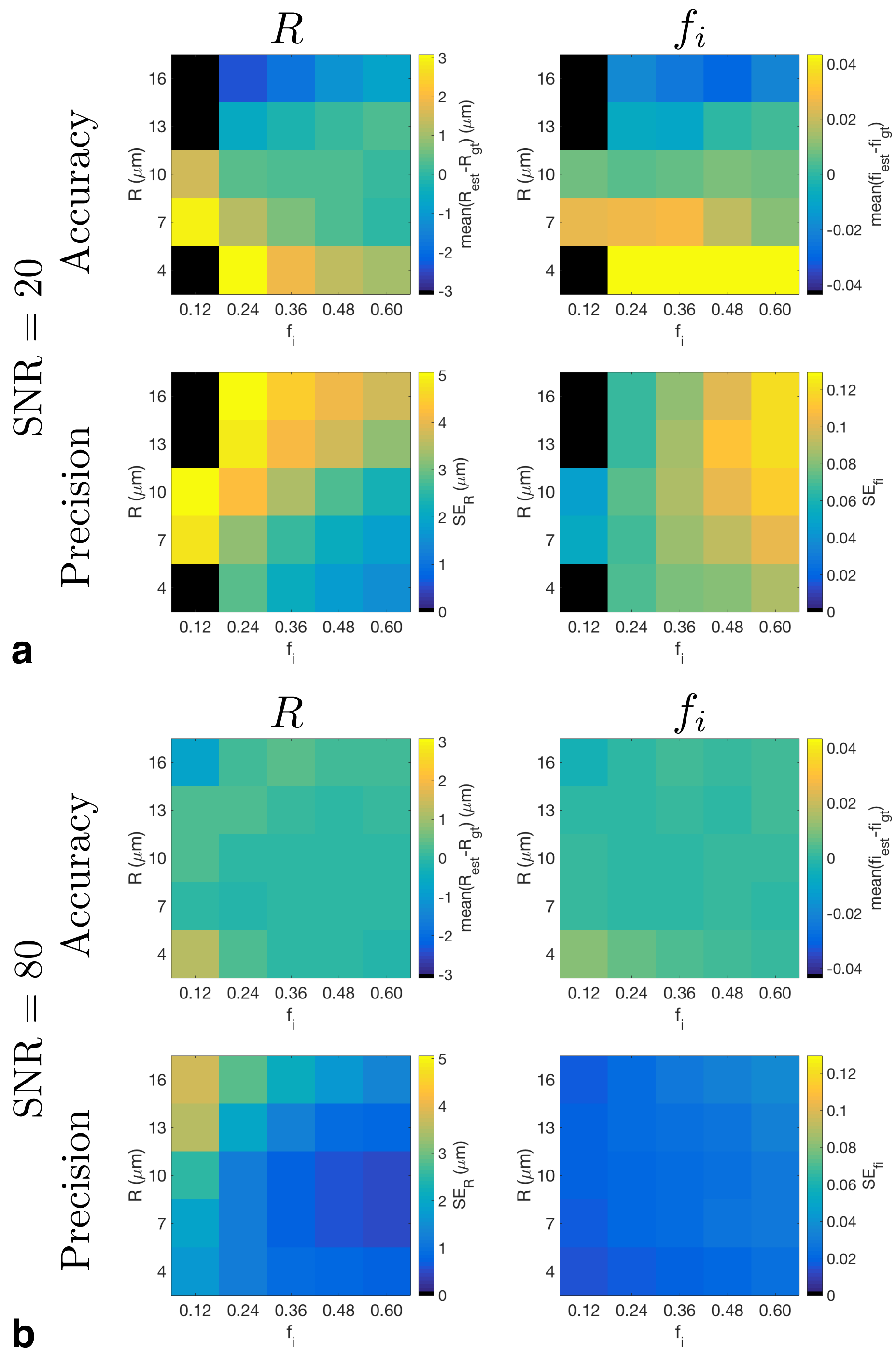

FIG. S1. Accuracy and precision of radius and intracellular volume fraction, using Non-opt$_{80}$, plotted as a function of ground truth R and f_i, for **(a)** SNR = 20, and **(b)** SNR = 80. Black points represent cases where more than 50% of the fits resulted in extreme values (within 1% of the fit constraints) for at least one parameter. Colour scales match those in Fig. 2 in main text.

Modelling asynchronous apoptosis: bimodal cell radius distribution

Simulations were performed to mimic asynchronous apoptosis, with some cells shrinking and some remaining the same size. Similar to the simulations in the main text, this analysis starts with a 'baseline' microstructure with $R = r$, $f_i = f$, $D_i = di$, and $D_e = de$. Two possible microstructural changes were then considered: (i) a simple mimic of asynchronous apoptosis, with a fraction of cells, p, remaining the same size, and the rest, $1 - p$, shrinking (with a cell volume decrease of 60%). The volume fractions of the resulting two cell populations, a and b, were calculated such that the cell density remained the same as at baseline. (ii) a simple mimic of complete cell death, with f_i decreasing from baseline, but R remaining constant. A specific example is shown in Figure S2a for $p = 0.50$, along with the equations used to control the microstructural changes. The case of $p = 0$ corresponds to the simulations presented in the main text; that is, where all cells shrink.

The effect of p on the SNR required for detecting ΔR and Δf_i was evaluated using the methods described in the main text, for a baseline of $R = 10\,\mu\text{m}$, $f_i = 0.60$, $D_i = 1\,\mu\text{m}^2/\text{ms}$, and $D_e = 2\,\mu\text{m}^2/\text{ms}$, with D-opt$_{80}$. Note that the generative signal model for change (i) here includes two cell radii, while all fitting was performed with the original model which only considers a single radius (Eqn. [1] in the main text). The biases associated with this are illustrated in Figure S2b, which shows the accuracy of R (top row) and f_i (bottom row) for the 'baseline' and two 'change' cases, as a function of SNR, for four p values (columns). The effect of having a generative model with two radii and a fitting model with a single radius is evident in the R estimates for case (i): as p increases, R estimates increase from $\sim 7.37\,\mu\text{m}$ to $\sim 10\,\mu\text{m}$ (gold crosses on top row of Fig. S2b). Here, the R estimates are approximately a weighted average of the two population radii, R_a and R_b, with weightings governed by the respective volume fractions. For f_i, the fitting model clearly cannot distinguish f_{i_a} and f_{i_b}, but the total restricted volume fraction, $f_{i_a} + f_{i_b}$, is estimated accurately (that is, gold crosses on bottom row of Fig. S2b are close to the dark orange dashed line).

Figure S3 plots the SNR required for detecting ΔR and Δf_i, as a function of p. For ΔR, the required SNR decreases slightly with increasing p, which is the result of a slight improvement in R precision as f_i increases, coupled with the fact that the total restricted volume fraction increases with p. For Δf_i, the required SNR increases with p, such that at $p = 0.75$ a higher SNR is needed to detect Δf_i than ΔR. This trend for f_i is due to the magnitude of Δf_i decreasing with increasing p, coupled with f_i precision becoming poorer as f_i increases.

While this suggests that detecting ΔR may be more feasible than detecting Δf_i, at least for $p >\sim 0.6$, this only considers the estimates' precision. As there is significant bias in the R estimates due to the bimodal radius distribution (see top row of Fig. S2b), the relevant radius decrease will not be estimated accurately; moreover, the estimate tends towards the baseline radius for high p, where it may be concluded that there is no change.

These simulations provide some insight into the application of single-radius models to data which come from a tissue with a distribution of radii, which will generally be the case experimentally (1). Further work could investigate fitting a radius distribution directly (2), though the SNR

requirements are likely to increase when fitting a model with more parameters.

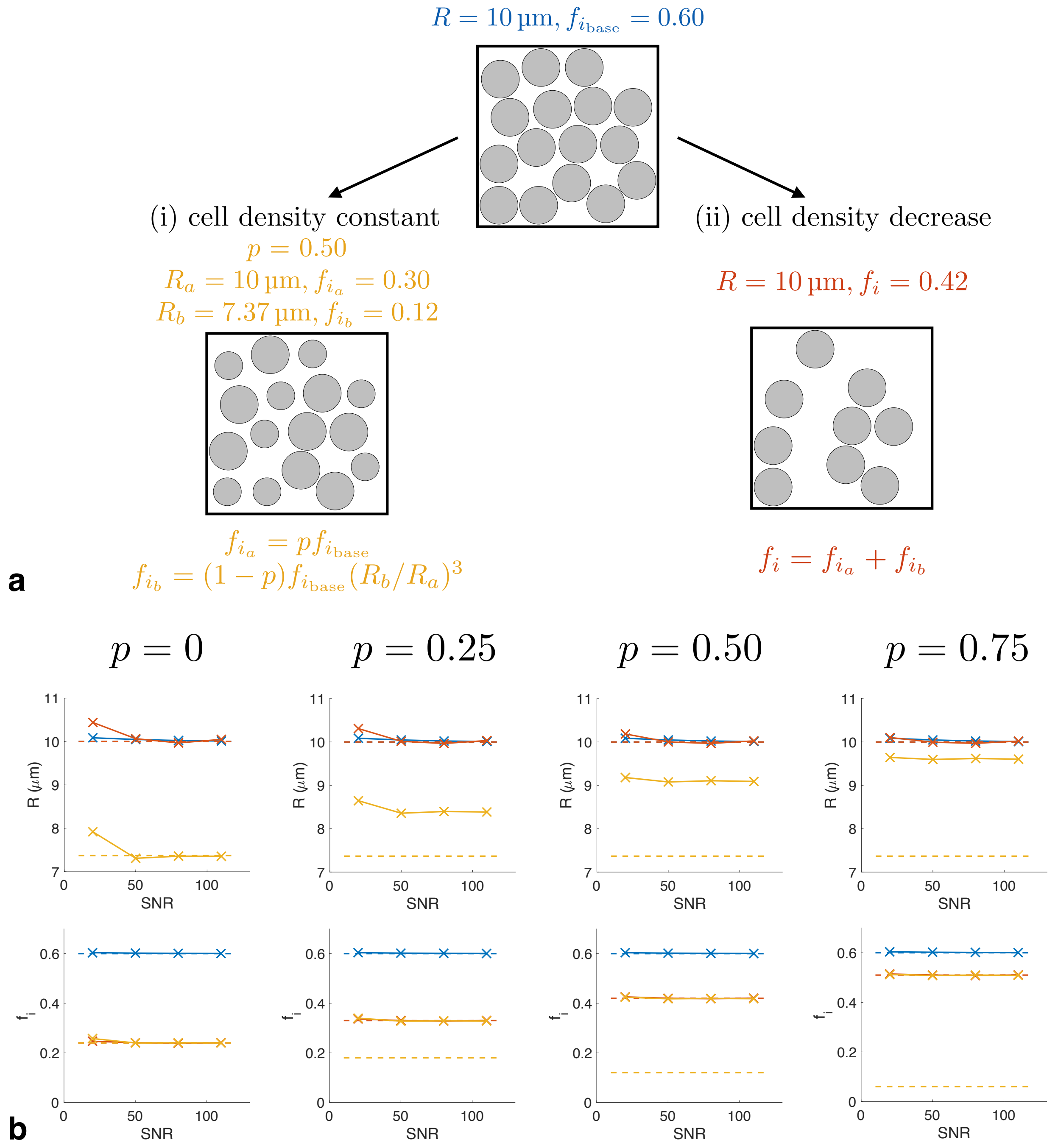

FIG. S2. Effects of having a bimodal cell radius distribution. **(a)** Schematic of baseline microstructure and changes, where a fraction, p, of cells remain the same size, and the rest, $1-p$, shrink. A specific example with $p = 0.50$ is used in the schematic, with the equations governing the changes shown at the bottom. The case of $p = 0$ corresponds to the simulations in the main text; that is, where all cells shrink. **(b)** Accuracy of R (top row) and f_i (bottom row) for three microstructures (colours), for $p = 0, 0.25, 0.50, 0.75$; dashed lines indicate ground truth, with the gold lines for R and f_i representing R_b and f_{i_b}, respectively.

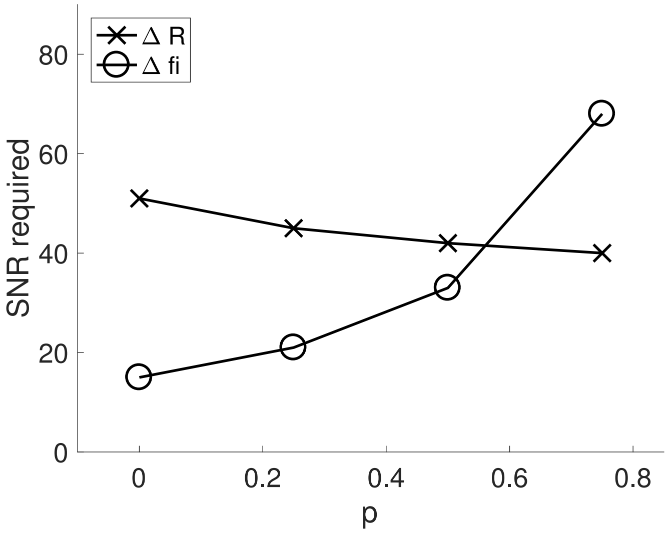

FIG. S3. SNR required for detecting ΔR (crosses) and Δf_i (circles), from changes (i) and (ii) respectively, as a function of p, the fraction of cells remaining the same size.

References

1. Alexander DC, Hubbard PL, Hall MG, Moore EA, Ptito M, Parker GJM, Dyrby TB. Orientationally invariant indices of axon diameter and density from diffusion MRI. NeuroImage 2010; 52:1374–1389.

2. Assaf Y, Blumenfeld-Katzir T, Yovel Y, Basser PJ. AxCaliber: a method for measuring axon diameter distribution from diffusion MRI. Magn Reson Med 2008;59:1347–1354.
